# Antioxidant, total phenolic contents and antinociceptive potential of *Teucrium stocksianum* methanolic extract in different animal models

**DOI:** 10.1186/1472-6882-14-181

**Published:** 2014-06-03

**Authors:** Syed Muhammad Mukarram Shah, Abdul Sadiq, Syed Muhammad Hassan Shah, Farhat Ullah

**Affiliations:** 1 Department of Pharmacy, University of Malakand, Chakdara Dir, KPK, Paksitan; 2 Department of Pharmacy, Sarhad University of Science and Information Technology, Peshawar, KPK, Pakistan

**Keywords:** *Teucrium stocksianum*, Antioxidant, Total phenolic contents, Analgesic

## Abstract

**Background:**

Oxidative stress and analgesia are connected with different pathological conditions. The drug candidates from synthetic sources are associated with various side effects; therefore, researchers are giving priority to find novel, effective and safe phytomedicines. *Teucrium* species possesses antioxidant, analgesic, anti-inflammatory and hepatoprotective activities. The essential oils of *Teucrium stocksianum* have shown strong antinociceptive potential. Our current study is designed to embark total phenolic content (TPC), antioxidant and antinociceptive potential of the methanolic extract of *Teucrium stocksianum* (METS).

**Method:**

Phytochemical composition was determined by using standard methods. Free radical scavenging potential and TPC of METS were assessed by using 2, 2-diphenyl-1-picryl-hydrazyl (DPPH) and Folin-Ciocalteu Reagent (FCR) respectively. Antinociceptive potential was determined by acetic acid induced abdominal writhing, formalin induced paw licking and tail immersion tests. Different test dose 50, 100 and 150 mg/kg body weight of METS were administered intra peritonealy (i.p) to various groups of mice for the evaluation of analgesic potential.

**Results:**

Phytochemical screening confirmed the presence of flavonoids, tannins, saponins, anthraquinone, steroid, phlobatannin, terpenoid, glycoside and reducing sugars. METS was found safe at a dose of 1000 mg/kg body weight. A concentration dependent free radical scavenging effect was observed with methanolic aerial parts extract of *Teucrium stocksianum* (MAPETS) and methanolic roots extracts of *Teucrium stocksianum* (MRETS). MAPETS and MRETS have shown highest antioxidant activity 91.72% and 86.19% respectively at 100 μg/ml. MAPETS was found more rich (115.32 mg of GAE/g of dry material) in TPC as compared to MAPETS (105.41 mg of GAE/g). METS demonstrated a dose dependent antinociceptive potential in different pain models, like in acetic acid, formalin and tail immersion showing 83.103%, 80.872% and 67.58% at a dose of 150 mg/kg, similar to acetylsalicylic acid (74.79%, 82.87%, 100 mg/kg) and Tramadol^R^ (74%, 30 mg/kg) respectively.

**Conclusion:**

Strong antioxidant potential and high TPCs are residing in the methanolic extract of *T. stocksianum*. METS showed analgesic potential in all models of nociception implying that both peripheral and central pathways of analgesia are involved. This might be due to the presence of various classes of phytochemicals in the plant extract.

## Background

The use of plants in curing various diseases is a re-explored field for the new research. Plants not only providing environmental survival to human beings but are also playing a vital role in the management of various diseases.

Medicinal plants synthesize secondary metabolites which are rich and novel sources of traditional and modern system of medicines. Herbal and natural products have a long known history in folk medicines. These products are safer, economical and can be used as parallel alternative to the modern synthetic drugs. The published literature available on medicinal plants has shown that phytochemicals can be used for the management of health problems like diabetes, cancer and infectious diseases [1,2].

Free radicals e.g. Reactive Oxygen Species (ROS) are toxic to the body when generated in high amounts as a result of oxidative stress. Although low levels of ROS are necessary for cells to carry out normal biochemical functions like cell signaling, apoptosis of defective cells etc. They are extremely reactive and at high levels during oxidative stress they may damage proteins, lipids and DNA molecules, which are responsible for various diseases like diabetes, cancer, Alzheimer’s and cardiovascular disease. Compounds obtained from natural sources can prevent toxic effects of the free radicals [3,4]. Medicinal plants possess antioxidant potential and can efficiently combat the free radicals produced inside the cell [5].

According to the International Association for the Study of Pain, it is, “an unpleasant sensory and emotional experience associated with actual or potential tissue damage, or described in terms of such damage” [6]. Plants have been used as antinociceptive agents since the pre-historic era. Currently, nonsteridal anti-inflammatory drugs (NSAIDs) and narcotics are the most prominent classes of pain killers. But there is marked risk of some serious side effects like renal, hepatic and gastrointestinal complications with the use of NSAIDs. While constipation and drug dependence issues are associated with narcotic analgesics [7,8].

Labiatae (Lamiaceae) is one of the largest families of flowering plants with about 220 genera and almost 4000 species worldwide [9]. Because of extensive species diversity and endemism in Labiatae some species are used in traditional medicine in Iran [10]. It has been reported that Teucrium species possesses antiseizure [11], antioxidant [12], acetylcholine easterase, butaryl choline esterease inhibition [13] and hepatoprotective activities [14]. *T. polium* possesses hypoglycemic [15], anti-inflammatory [16] and antinociceptive activities [17]. The therapeutic benefit of medicinal plants is often attributed to their antioxidant properties [18].

Different groups of researchers have reported various pharmacological activities of *T. stocksianum* like cytotoxic and anthelmintic [19]. Gul Rahim et al. have reported different folkloric uses of this plant from Pakistan [20].

Looking to the pharmacological potential of *T. stocksianum* we designed a research project to evaluate the crude methanolic extract of *T. stocksianum* for antioxidant, total phenolic contents and antinociceptive potential.

## Methods

### Plant material

Whole plant of *T. stocksianum* (TS) has been collected from Marghuzar valley of District Swat Khyber Pakhtunkhwa (KPK), Pakistan in the month of May 2012. The plant was authenticated by Dr. Nasrullah in the Department of Botany, University of Malakand, Pakistan. A voucher specimen (H.UOM.BG.199) of the plant was deposited in the University Herbarium for future reference.

### Preparation of plant extract

The plant was air dried under shade, cut into small pieces and then pulverized to coarse powder. About one kilogram of the powdered plant material was macerated with methanol (80%) for two days. The methanolic extract was concentrated at reduced pressure at 45°C temperature using rotary evaporator. The concentrated methanolic extract was stored in glass container in refrigerator for future use. The same procedure was repeated for the aerial parts and roots separately according to the design of our experimental procedure.

### Experimental animal

Swiss albino mice of either sex, same age and 18-22 g weight were used in the present study. All the animals were housed in appropriate cages at standard controlled laboratory condition (23°C, 12 h light/dark cycle). Water and food were easily available to all experimental animals during acclimatization period. Food was withdrawn from all animals 18 hrs before starting the experiment. The animals were randomly divided into five groups (*n* = 6). This study was approved by the ethical committee of Department of Pharmacy, University of Malakand according to animals Bye-Laws 2008 (Scientific procedure Issue-1).

### Chemicals and drugs

All chemicals used in this study were of analytical grade purchased from Merck, Pakistan and Tramadol^R^ was obtained from Searle Pakistan Ltd.

### Statistics and calculations

The results obtained were expressed as mean ± SEM (Standard error of mean) and Standard deviation (SD). One-way ANOVA followed by multiple comparison Dunnet test was applied for comparison of the test group with control. Differences with *P ≤* 0.05 were considered significant.

### Acute toxicity

Acute toxicity test was conducted for METS. Four groups of Swiss albino mice of either sex (*n* = 6) were treated with various test doses, i.e. 250, 500 and 1000 mg/kg intra peritoneal. The control group received 1% tween-80 (10 ml/kg). Animals were constantly observed for any gross effect for initial 4 h and then the number of dead animals were recorded after 24 h [21].

### Phytochemical composition

Phytochemical screening of the METS was carried out using standard procedures [22,23].

### Antioxidant potential

#### DPPH radical scavenging potential

DPPH (1,1-diphenyl-2-picryl-hydrazyl) is a stable free radical most commonly used for the evaluation of radical scavenging potential of extract/fractions of phytomedicine. Procedure reported by Blois and Yildirim was followed with slight modifications [24,25]. DPPH radical solution was prepared in methanol (24 mg/100 ml) and 1 ml of this solution was added to 1 ml of methanolic solution of sample containing 20, 40, 60, 80 and 100 μg of the extract respectively. Methanol was used as negative control while Gallic acid, Quercetin and Ascorbic acid served as positive controls. The samples and control solutions were incubated in dark for 30 min at controlled temperature (20–25°C). After incubation bleaching of the purple coloured methanolic solution of DPPH was observed. Absorbance of samples, control and standard were measured at 517 nm. Percent radical scavenging activities (%RSA) by DPPH free radical was calculated as follow.

$$\%\mathrm{RSA}=\frac{\text{Control}\;\text{absorbance}-\text{Sample}\;\text{absorbance}\;}{\text{Control}\;\text{absorbance}}\;\times \;100$$

The test of each sample was performed in triplicate and the results were expressed as mean values ± standard deviations. Inhibitory concentration (IC_50_) was calculated.

#### Determination of total phenolic contents

The antioxidant activity of medicinal plants is mostly due to the presence of phenol group (s) containing phytochemicals [26]. Total phenolic contents were estimated according to the procedure reported by Singleton et al. [27]. Calculated quantity of the methanolic extract was dissolved in methanol in order to get 500 *μ*g concentrations. To 1 ml of this solution 45 ml of distilled water and 1 ml of Folin-Ciocalteu Reagent (FCR) was mixed in a volumetric flask. Then after brief incubation (3 min) 3 ml of Na_2_CO_3_ (20%) solution was added to the mixture to make a final volume 50 ml. Afterwards, mixture was gently shacked for 2 h at controlled temperature (20–30°C) and absorbance was taken at 760 nm. Assay was performed in triplicate. The results were expressed as milligrams of gallic acid equivalent gram of dry weight (mg GAE/g dw) and were calculated from the standard curve, using the following formula;

A=1156C+0189

Where “A” is the absorbance of standard and sample and “C” is the Gallic acid equivalent (mg/g).

### Antinociceptive activity

#### Acetic acid induced writhing test

In this study five groups (*n* = 6) were selected. The test groups received METS at a dose of 50, 100 and 200 (mg/kg, b.w, i.p) respectively. Control group was treated with 1% Tween 80 solution (10 ml/kg, b.w, i.p). Standard group received acetylsalicylic acid (100 mg/kg, b.w, s.c). All the groups were treated with test sample (10 ml/kg, 0.6%, i.p) after 30 min of writhes induction. The number of abdominal writhes was counted for 20 min after 5 min of acetic acid administration. Percent inhibition was calculated by comparing the results of METS with the control group [28].

#### Formalin induced paw licking test

All mice were transferred to the observation chamber 10 min before the experiment for acclimatization to the new environment. Mice were divided into five groups (*n* = 6). Control group was treated with 1% Tween 80 solution (10 ml/kg, i.p). Test groups received METS 50, 100 and 200 mg/kg (i.p). Standard group was treated with acetylsalicylic acid (100 mg/kg, s.c). After 30 min of the drug administration, pain was induced in all mice by injecting 1% formalin solution (20 μl) into the right hind paw through intraplantar route. Formalin (1%) solution was prepared by diluting commercial formalin solution (10%) with normal saline. The total number of licking the injected paw were observed during 0-5 min (Neurogenic, initial phase) and 20-30 min (Inflammatory, late phase) after formalin injection. Special measures were taken in order to avoid any possible disturbance during experiments that may affect the animal’s response.

#### Tail immersion test

Five groups of six mice (*n* = 6) in each were selected for this study. Group 1 (control) received Tween 80 (1%, 10 ml/kg, b.w, i.p), group 2, 3 and 4 were treated with 50, 100 and 200 mg/kg, b.w, i.p METS respectively. Group 5 was treated with standard drug Tramadol^R^ (30 mg/kg). Pain reactions were induced by dipping the animal’s tail (2-5 cm) in a pot of water maintained at 54 ± 0.5°C. The reaction time (in seconds) is the time taken by the animal to withdraw the tail from worm water. The reaction time was noted after 30 min of the test drug administration. In order to avoid tissue damage a 30 seconds cut-off time was maintained at 54 ± 0.5°C. The reaction time was observed at 0, 30, 60, 90 and 120 min after drug administration [29]. Naloxone was administered in a dose of 0.5 mg/kg body weight to observe whether the analgesia is antagonized or not.

## Results

### Acute toxicity

In this experiment METS was found safe at different test doses (250, 500 and 1000 mg/kg b.w, i.p). All the test animals were found normal. There were no signs of convulsions and any other gross effect during 24 h test time, which indicates low toxicity profile of METS.

### Phytochemical composition

Preliminary phytochemical screening of METS indicated the presence of various classes of secondary metabolites like flavonoids, tannins, saponins, anthraquinones, steroids, phlobatannins, terpenoids, glycosides and reducing sugars.

### Antioxidant potential

#### DPPH radical scavenging potential

A concentration dependent free radical scavenging effect was observed with methanolic extract. The antioxidant activity of five different concentrations (20, 40, 60, 80 and 100 μg/) was 82.43%, 85.93%, 87.38%, 89.38% and 91.72% respectively with IC_50_ value of 12.5 μg/ml.

Values are expressed as the mean ± S D. (mg GAE/g dw); milligrams of galic acid equivalent per gram of dry weight of plant material.

MRETS also exhibited a concentration depended radical scavenging effect. Different concentrations 100, 80, 60, 40, and 20 μg/ml have shown 86.19%, 77.89%, 68.54%, 59.96% and 46.66% radical scavenging activity respectively with IC_50_ value 24.5 μg/ml. The %RSA value of the extract was also compared with that of Quercetin (Table 1).

**Table 1:** **Comparison of %RSA, TPC and IC**_**50 **_**values of MAPETS and MRETS with Quercetin (Q) Values are expressed as the mean ± S D**

**TEST Solutions**		**Comparison of %RSA (Con μg/ml)**				**TPC (mg GAE/g dw)**	**IC**_**50 **_**(Con μg/ml)**
**Sample/standard**	**20**	**40**	**60**	**80**	**100**		
MAPETS	82.58 ± 0.93***	85.68 ± 0.57***	87.58 ± 0.60***	89.16 ± 1.36***	91.6 ± 0.90***	115.55 ± 0.74	12.5
MRETS	46.55 ± 1.11***	59.91 ± 019***	68.51 ± 0.60***	77.69 ± 0.86***	86.3 ± 0.4***6	104.9 ± 2.16	24.5
Q	94.95 ± 0.86	95.35 ± 0.57	96.48 ± 0.53	97.55 ± 0.58	98.2 ± 0.81	_____	10.2

Results of multiple comparison Dunnetts test in one-way ANOVA; Groups significantly different *** p ≤ 0.001 when compared to positive control group. ***p ≤ 0.001, **p ≤ 0.01 and *p ≤ 0.05 vs. control group (n = 6).

#### Total phenolic contents

Table 1 shows that crude methanolic extract of the aerial parts was composed of high quantity of total phenolic contents as compared to the root extract i.e. 115.32 mg and 105.41 mg of gallic acid equivalent per gram (GAE/g) of dry material respectively. The difference in the phenolic contents may be due to the presence of essential oils in the aerial parts of the plant.

#### Acetic acid induced writhing test

The antinociceptive potential of different doses of the METS are shown in Table 2. In control group maximum number of abdominal writhes during 20 min test period was 60.166 ± 4.167. The test groups treated with different concentrations of METS (50, 100 and 150 mg/kg b.w), produced a significant (*p* < 0.001) and a dose dependent inhibition of abdominal writhes when compared to control group. Maximum writhes inhibition (83.103%) was noted at highest concentration (150 mg/kg). The standard drug (Acetylsalicylic acid, 100 mg/kg) produced 74.793% writhes inhibition when compared to control group.

**Table 2:** Antinociceptive effect of METS 50, 100 and 150 mg/kg in acetic acid induced abdominal writhing test

**Samples**	**Dose**	**Number of writhes**	**% inhibition**
1% tween 80	10 ml/kg	60.166 ± 4.167	**-**
METS	50 mg/kg	30.166 ± 4.070	49.862***
	100 mg/kg	22.833 ± 2.786	62.05***
	150 mg/kg	10.166 ± 2.786	83.103***
Acetylsalicylic acid	100 mg/kg	15.166 ± 3.656	74.793

Results of multiple comparison Dunnetts test in one-way ANOVA; Groups significantly different *** p ≤ 0.001 when compared to positive control group. Percent inhibition was calculated in comparison to control group. *F =* 187.523*, d.f. =* 29. ED50 for analgesic effect of METS was 50.68 mg/kg (b.w). ***p ≤ 0.001, **p ≤ 0.01 and *p ≤ 0.05 *vs.* control group (n = 6).

#### Formalin induced paw licking

MAPETS showed a dose dependent relationship in Phase I (neurogenic) as well as in Phase II (inflammatory) of formalin induced pain. In Phase I the extract produced a significant inhibition (19.787%, *p* < 0.05) at a dose of 150 mg/kg compared to control. In phase II the extract produced a significant inhibition (50.275%, *p* < 0.001), (77.348%, *p* < 0.01), (80.663%, *p* < 0.05) at a dose of 50, 100 and 150 mg/kg as shown in Table 3.

**Table 3:** Effect of MAPETS 50, 100 and 150 mg/kg in Formalin induced paw licking

**Samples**	**Dose**	**Phase I**		**Phase II**	
		**No of paw licking**	**% inhibition**	**No of paw licking**	**% inhibition**
1% Tween 80	10 ml/kg	47.166 ± 2.85	**____**	30.166 ± 1.70	**____**
MAPETS	50 mg/kg	42.666 ± 1.87	09.542	15.166 ± 1.74***	50.275
	100 mg/kg	39.166 ± 3.00	16.961	6.333 ± 0.98**	77.348
	150 mg/kg	37.833 ± 2.64*	19.787	5.833 ± 0.90*	80.663
Acetylsalicylic acid	100 mg/kg	40.833 ± 2.49	____	2.833 ± 0.60	82.872

Values expressed as mean ± SEM. Percent inhibition was calculated in comparison to the control group. ***p ≤ 0.001, **p ≤ 0.01 and *p ≤ 0.05 *vs.* control group (n = 6).

#### Tail immersion test

In this procedure different concentrations (50, 100 and 150 mg/kg) of METS were tested in various groups of mice. The reaction time of animals against heat stimulus was recorded. The analgesic effect of METS was non significant in all the test doses. The highest effect (67.58%) of the extract was noted at 60 min at a dose of 150 mg/kg compared to control group. Moreover, the analgesic effect of Tramadol^R^ (30 mg/kg) and METS (150 mg/kg) was antagonized by naloxone as shown in Table 4.

**Table 4:** Effect of METS 50, 100 and 150 mg/kg in Tail immersion test

**Samples**	**Dose mg/kg**	**Tail withdrawing time in sec**				
		**0 min**	**30 min**	**60 min**	**90 min**	**120 min**
1% tween 80	10 ml/kg	3.45 ± 0.26	3.48 ± 0.19^ns^	3.27 ± 0.032^ns^	3.48 ± 0.20^ns^	3.57 ± 0.19
METS	50 mg/kg	3.48 ± 0.19	3.57 ± 0.19^ns^	4.11 ± 0.12^ns^	4.16 ± 0.12^ns^	4.09 ± 0.11^ns^
	100 mg/kg	3.53 ± 0.18	4.25 ± 0.04^ns^	4.49 ± 0.13^ns^	4.39 ± 0.08^ns^	4.29 ± 0.04^ns^
	150 mg/kg	3.57 ± 0.19	4.82 ± 0.04^ns^	5.48 ± 0.02^ns^	5.27 ± 0.05^ns^	5.18 ± 0.06^ns^
Tramadol^R^	30 mg/kg	3.48 ± 0.20	4.99 ± 0.33	5.69 ± 0.02	5.49 ± 0.06^ns^	5.38 ± 0.05^ns^
Antinociceptive effect of METS and Tramadol^R^ antagonized by Naloxone						
METS	150 mg/kg	3.52 ± 0.02 ^ns^	2.64 ± 0.01^ns^	2.87 ± 0.02^ns^	2.73 ± 0.01^ns^	2.69 ± 0.01^ns^
Tramadol^R^	30 mg/kg	3.50 ± 0.02	2.35 ± 0.02	2.69 ± 0.01	2.75 ± 0.02	2.65 ± 0.02

^ns^Values statistically non significant when compared to control using one way ANOVA followed by Dunnett’s multiple comparison test.

## Discussion

*Teucrium* genus has been extensively studied and proved to be a useful medicinal herb. A diverse array of pharmacological activities have been reported from this genus. In our previous work, we reported the chemical composition and antinociceptive potential of the essential oil of *T. stocksianum*[30]. In the current study we investigated antioxidant, TPC and antinociceptive potential of the crude methanolic extract of *T. stocksianum*.

Geographical location and temperature variations are very important for production of secondary metabolites which actually possess therapeutic properties (M.A. Couceiro, A. Cristina Figueiredo). We are reporting data of *Teucrium stocksianum* collected in the cold hilly area of North-West of Pakistan i,e Swat while Radhakrishnan et al. have reported data from plant collected from the hot environment of UAE (Radhakrishnan). Our results are different from results reported by the other study.

Living organisms during normal metabolism produce reactive oxygen species (ROS) which are responsible for a number of diseases such as rheumatic joint inflammation, Alzheimer’s, Parkinson’s, diabetes, hypertension, atherosclerosis and oxidative stress [31]. It is well documented that flavonoids possess significant antioxidant potential due the presence of phenolic hydroxyl groups [26]. Our results show that the extract of the aerial parts of TS possesses strong antioxidant potential and high quantity of TPC (91.66%, 115.55 mg GAE/g dw) as compared to roots (86.34%, 104.9 mg GAE/g dw) which might be due to the presence of high quantity of total phenolic contents and flavonoids. Moreover phytochemical screening of METS has also shown the presence of flavonoids. Many studies are available which relate analgesic, anti-inflammatory and antioxidant activities with flavonoids by interacting with prostaglandins and superoxides [32].

Acetic acid causes abdominal constrictions by releasing certain endogenous chemical substances like histamine, serotonin, bradykinin and different prostaglandins which stimulate the pain sensitive neurons. These neurons are sensitive to pain relieving drugs like non steroidal anti-inflammatory drugs (NSAIDs) and narcotics [33,34]. In acetic acid induced writhing test, METS exhibited significant results. At 50, 100 and 150 mg/kg doses the abdominal constrictions were reduced to 49.86%, 62.05% and 83.10% (*p* < 0.001) respectively as shown in Table 2. It has been found that METS possesses strong antinociceptive potential as compared to the methanolic extract of *T. polium* (65.44%, 225 mg/kg) and aqueous extract of *T. chamaedrys* (76.4%, 200 mg/kg). It has been reported that the essential oil of TS possesses significant 93.97% (*p* < 0.001) antinociceptive activity at a very low dose (80 mg/kg) [30] as compared to the methanolic extract of the same plant.

It has been postulated by Collier et al. that acetic acid causes an indirect stimulation of the nociceptive neurons sensitive to the opioids and NSAIDs by inducing the releases of various endogenous mediators [35]. Based on our current findings, the antinociceptive activity of METS is possibly due to inhibition of response or release of endogenous noxious mediators that causes activation and sensitization of peripheral nociceptors. Therefore, the results of acetic acid induced abdominal constrictions are not sufficient to understand the exact antinociceptive mechanism, that the effect produced by the extract was mediated by central or peripheral pathways [36].

In order to understand the mechanism of analgesia, formalin induced pain model was used [37]. This is a biphasic antinociceptive procedure. The centrally acting drug inhibits both phase I and phase II while the peripherally acting drugs can only inhibit phase II of formalin induced pain model [38]. In this study it was observed that the METS significantly decreased the number of paw licking in both phases of formalin induced pain, indicating that the extract caused the analgesic effect through central mechanism. It was found that the extract at a dose of 150 mg/kg was highly effective producing 80.663% inhibition in phase II, suggesting a strong peripheral effect (Table 4).

Tail immersion test is a specific central procedure. This test has shown that mu (μ) opioids receptors were involved in antinociceptive effect rather than kappa (k) or delta receptors [39]. Literature shows that descending inhibition pathways and opioidergic system involving serotonergic and noradrenergic systems play a vital role in pain regulation at spinal and supraspinal level [40,41]. The METS has significantly increased the latency time (%) at a dose of 150 mg/kg. Maximum effect for all test doses of METS was recorded after 60 min which persists upto 120 min. Naloxone an opioid receptor antagonist, when applied reversed profoundly the analgesic effect of METS after 30 min of administration. This shows that the antinociceptive potential of the extract is due to activation of the opioid receptors.

In conclusion, the methanolic extract of *T. stocksianum* possesses antioxidant and antinociceptive potentials. According to our findings both peripheral and central pathways are involved in relief of pain.

## Competing interests

The authors have declared that they have no conflict of interest.

## Authors’ contributions

SMMS performed all the experiments, FU and SMHS did literature survey, AS, FU and SMMS prepared the manuscript. Before submission all the authors have checked and approved the final manuscript.

## Pre-publication history

The pre-publication history for this paper can be accessed here:

http://www.biomedcentral.com/1472-6882/14/181/prepub
